# Recent Advances in the Synthesis, Characterization and Application of Zn^+^‐containing Heterogeneous Catalysts

**DOI:** 10.1002/advs.201500424

**Published:** 2016-02-03

**Authors:** Guangbo Chen, Yufei Zhao, Lu Shang, Geoffrey I. N. Waterhouse, Xiaofeng Kang, Li‐Zhu Wu, Chen‐Ho Tung, Tierui Zhang

**Affiliations:** ^1^ Key Laboratory of Synthetic and Natural Functional Molecule Chemistry of the Ministry of EducationCollege of Chemistry and Materials ScienceNorthwest UniversityXi'an710069P.R. China; ^2^ Key Laboratory of Photochemical Conversion and Optoelectronic MaterialsTechnical Institute of Physics and ChemistryChinese Academy of SciencesBeijing100190P.R. China; ^3^ School of Chemical SciencesThe University of AucklandAuckland1142New Zealand

**Keywords:** Zn^+^, layered double hydroxide, zeolite, methane conversion, CO_2_ photoreduction

## Abstract

Monovalent Zn^+^ (3d^10^4s^1^) systems possess a special electronic structure that can be exploited in heterogeneous catalysis and photocatalysis, though it remains challenge to synthesize Zn^+^‐containing materials. By careful design, Zn^+^‐related species can be synthesized in zeolite and layered double hydroxide systems, which in turn exhibit excellent catalytic potential in methane, CO and CO_2_ activation. Furthermore, by utilizing advanced characterization tools, including electron spin resonance, X‐ray absorption fine structure and density functional theory calculations, the formation mechanism of the Zn^+^ species and their structure‐performance relationships can be understood. Such advanced characterization tools guide the rational design of high‐performance Zn^+^‐containing catalysts for efficient energy conversion.

## 1 Introduction

This is an open access article under the terms of the Creative Commons Attribution License, which permits use, distribution and reproduction in any medium, provided the original work is properly cited.

Zinc (Z = 30) compounds have been extensively investigated for utilization in therapeutic activity, drug and gene delivery, and in nanotech applications including photocatalysis and UV sensors, all with promising outcomes.^1^, ^2^ Metallic Zn has the 3d^10^4s^2^ ground state electron configuration, with the filled *d*‐orbitals of Zn being very stable and seldom taking part in bonding in Zn compounds. Metallic Zn is easily oxidized, losing its two 4s electrons to form the divalent Zn^2+^ cation (3d^10^). Thus the main oxidation states of Zn are 0 and +2. Recent calculations suggest that it is actually impossible to get a stable Zn^3+^ compound.^3^ However, it is possible to synthesize monovalent Zn^+^ (3d^10^4s^1^)‐containing compounds with special electron structure, which demonstrate interesting functional properties such as catalytic conversions of methane, CO and CO_2_ relative to related divalent Zn^2+^ compounds (ZnO,^4^, ^5^ ZnS,^6^ ZnSe,^7^ Zn‐containing spinels,^8^ Zn^2+^‐complex,^9^ Zn^2+^‐containing enzymes,^10^ etc.) which have been extensively studied mainly for sensors, solar cells, optoelectronic devices, gene therapy and photodegradation of organic pollutants. However, the synthesis of monovalent Zn^+^ compounds with an unpaired *s*‐orbital electron is a challenge, and recently has attracted much attention.

Very recently, nanosized materials containing coordinatively unsaturated metals have been successfully synthesized which show distinct catalytic activity and selectivity due to their unique physical, chemical and electronic properties. Experimental and theoretical investigations have demonstrated that coordinatively unsaturated metals ions in unusual oxidation states such as Fe^2+^,^11^, ^12^ Ti^3+^,^13^ Ni^3+^ 14 and Co^3+^ 15 can act as highly effective electron donors or acceptors, facilitating electron transfer to or from reactants, resulting in improved catalytic performance. In the past few decades, considerable research effort has been directed towards the development of monovalent Zn^+^‐containing compounds, due in part to their excellent catalytic activity in energy conversion applications. To the best of our knowledge, no recent review paper has been published describing the synthesis and applications of Zn^+^‐containing compounds. We believe it is timely to review this field.

**Figure 1:**
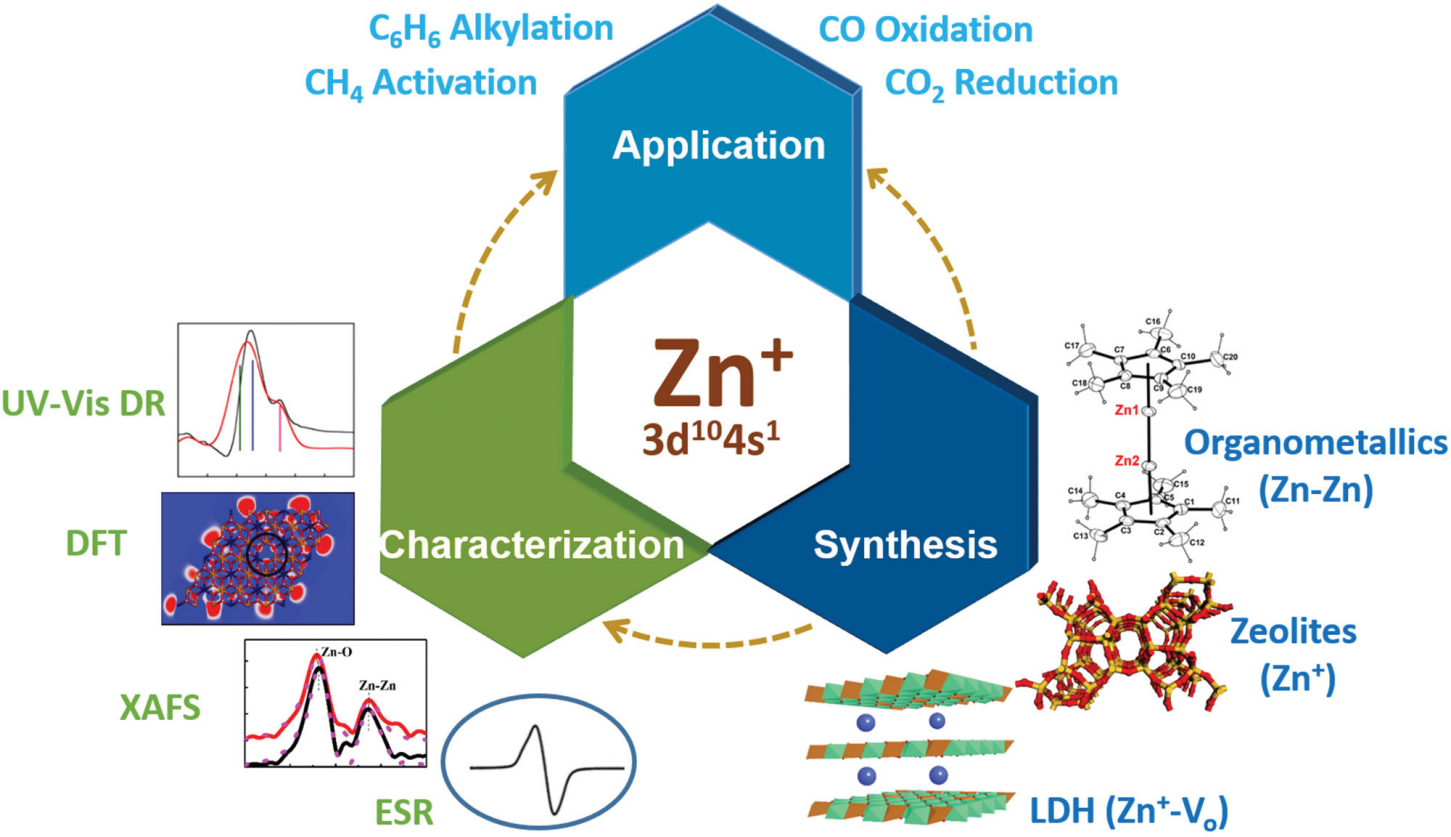
Schematic illustration of the synthesis, characterization and application of Zn^+^‐containing heterogeneous catalysts.

In this review, we provide an overview of recent developments related to the synthesis, characterization and application of Zn^+^‐containing heterogeneous catalysts (**Figure** **1**). Zn–Zn bonded organometallic compounds are also introduced in the synthesis section, despite there being no obvious application of such compounds in heterogeneous catalysis to date. However, the inclusion of Zn–Zn bonded organometallic compounds guides possible future synthesis pathways to Zn^+^ compounds. The characterization techniques discussed include electron spin resonance (ESR), X‐ray absorption and diffraction structure analysis, density functional theory (DFT) calculations and UV–vis diffuse reflectance (UV–vis DR) spectroscopy, and their value in probing Zn^+^ compound structure is critically discussed. Finally, the potential applications of monovalent Zn^+^‐compounds in energy conversion are highlighted. Particular emphasis is placed on the exploration of structure‐activity relationships through combining experimental techniques with theoretical calculations, thereby allowing the rational and smart design of highly efficient Zn^+^‐containing heterogeneous catalysts for different applications.

## 2 Synthesis of Zn^+^‐Containing Materials

Divalent Zn^2+^ cations are readily formed from metallic Zn by losing two 4s electrons. In order to obtain compounds containing monovalent Zn^+^ cations (3d^10^4s^1^), many different methods have been attempted. Until the middle of the last century, monovalent Zn^+^ cations could only be obtained in the gas phase under harsh physical conditions such as γ‐irradiation,^16^ electron impact ionization^17^ and glow discharge.^18^ Such gas phase Zn^+^ species were of little use for practical applications. A new class of Zn^+^ complexes, including Zn^+^(H_2_O)*_n_* and Zn^+^(H_2_)*_n_*, were also obtained in an external laser vaporization source,^19^ though the physical properties and chemical reactions of these complexes remained unexplored for a long time.^20^, ^21^, ^22^, ^23^

### 2.1 Synthesis of Zn–Zn Bonded Organometallics

**Figure 2:**
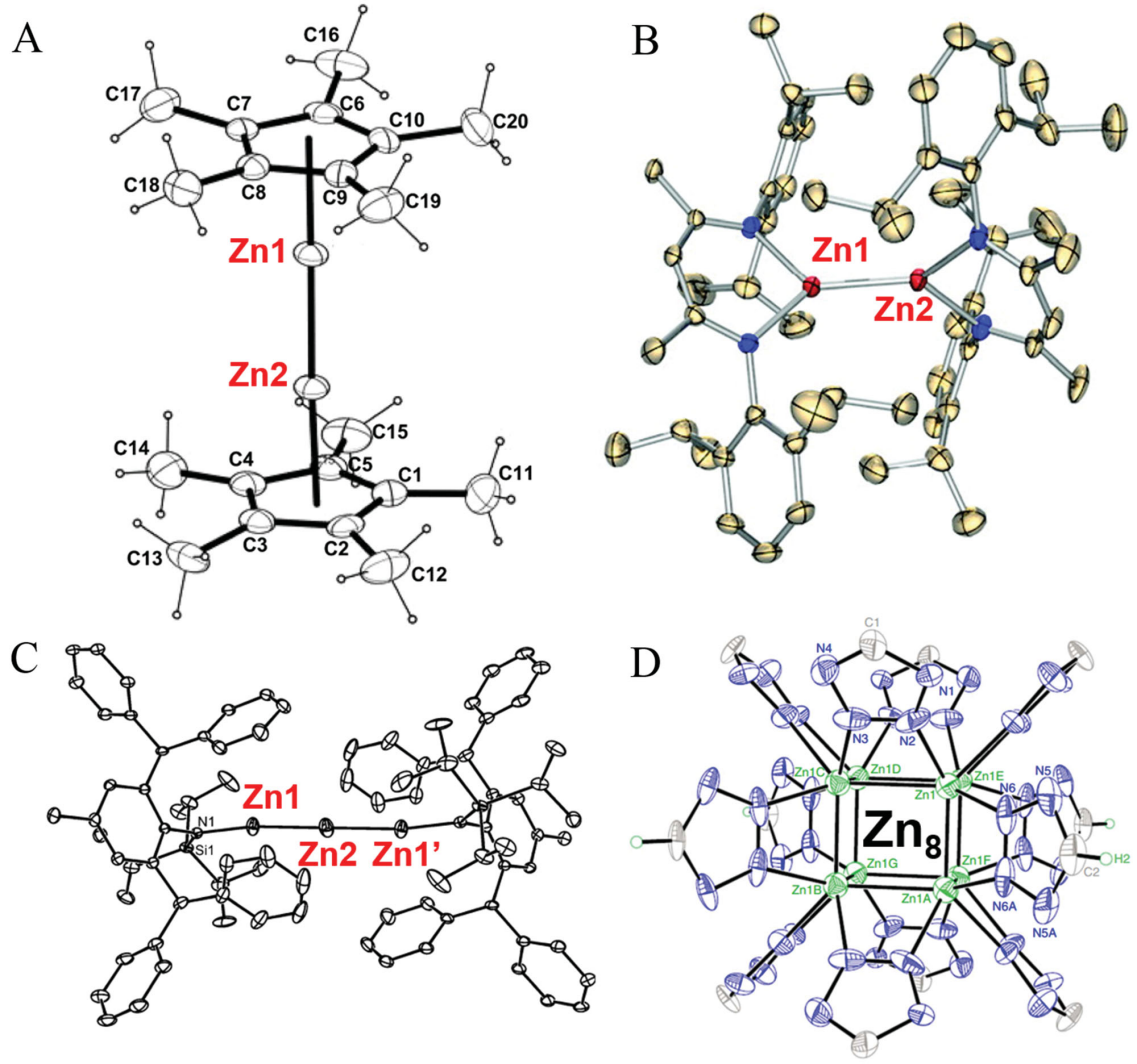
Molecular structures of Zn–Zn bonded structures, A) Zn_2_(η^5^‐C_5_Me_5_)_2_, B) Zn_2_([(2,6‐*i*‐Pr_2_C_6_H_3_)N(Me)C]_2_‐CH)_2_, C) [LZn^I^Zn^0^Zn^I^L] (L = bulky amide) and D) [Zn^I^_8_(HL)_4_(L)_8_]^12–^ (L = tetrazole dianion). A) Reproduced with permission.^25^ Copyright 2004, American Association for the Advancement of Science. B) Reproduced with permission.^26^ Copyright 2005, American Chemistry Society. C) Reproduced with permission.^28^ D) Reproduced with permission.^29^ Copyright 2015, Nature Publishing Group.

The first report about the chemical synthesis of monovalent Zn^+^ compounds involved mixing metallic Zn with fused zinc chloride at 500–700 °C, leading to the formation of Zn_2_^2+^ species with a Zn–Zn bond.^24^ In 2004, Carmona's group reported the successful synthesis of the organometallic half‐sandwich compound decamethyldizincocene (Zn_2_(η^5^‐C_5_Me_5_)_2_), containing a central subvalent Zn–Zn bond with both the Zn atoms formally in the +1 oxidation state (**Figure** **2**A).^25^ This discovery opened a new chapter in organometallic chemistry, namely the synthesis of Zn–Zn bonded complexes. Subsequently, such molecular compounds stabilized by a variety of sterically demanding, often chelating, organic ligands have been synthesized and characterized, as showed in Figure 2B for an example.^26^ In 2012, Roesky's group reviewed the synthesis, reactivity and applications of Zn–Zn bonded complexes.^27^ Very recently, the family of subvalent Zn–Zn bonded structures has been extended to include mixed‐valence linear tri‐zinc complex [LZn^I^Zn^0^Zn^I^L] (L = bulky amide) (Figure 2C)^28^ and multicenter‐bonded polyzinc compounds like [Zn^I^_8_(HL)_4_(L)_8_]^12−^ (L = tetrazole dianion) which has an unusual cubic cluster core, containing monovalent Zn^+^ ions and short Zn–Zn bonds (Figure 2D).^29^ However, practical applications for such Zn–Zn bonded compounds are rarely reported due to the low synthesis yields and their high sensitivity to air or water. These Zn^+^‐based compounds loaded on various supports could show interesting potential in heterogeneous catalysis in next stage.

### 2.2 Synthesis of Zn^+^‐Containing Sites in Zeolites

**Figure 3:**
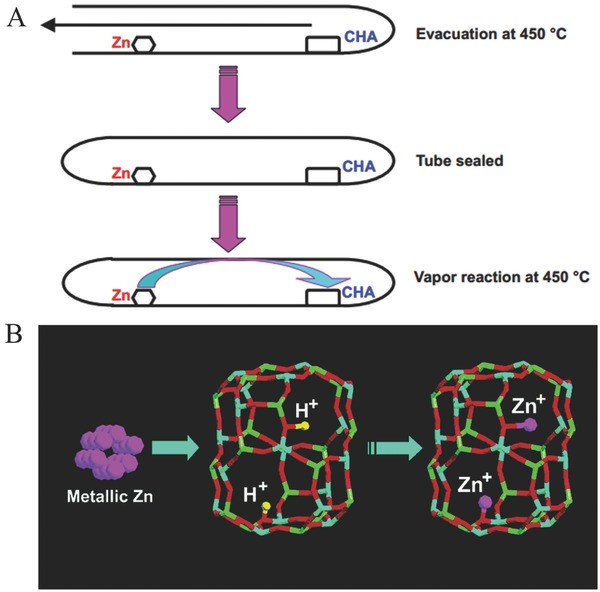
A) Preparation procedure of Zn@SAPO‐CHA. B) Schematic representation of the formation of two mononuclear Zn^+^ cations in one CHA cage of SAPO‐CHA. Reproduced with permission.^32^ Copyright 2003, American Chemical Society.

Zeolites, due to their inherent well‐defined pore structure and electronic properties, have received huge attention in solid‐state chemistry and materials science. Exposure of zeolites to metal vapor is an effective approach for the preparation of metal‐containing zeolites.^30^ The isolated acidic sites (bridging OH groups) and host‐guest nature of zeolites make them strong candidates for realizing Zn^+^ species, through the reaction of metallic Zn with acidic sites. By this route, Zn_2_^2+^ species were successfully formed in the Zn exchanged zeolite‐Y by a chemical vapor deposition technique.^31^ Subsequently, Chen's group firstly synthesized mononuclear monovalent Zn^+^ in microporous crystalline silicoaluminophosphate molecular sieves possessing the chabazite structure (SAPO‐CHA) (**Figure** **3**).^32^ The protonated acidic sites in the cages of microporous SAPO‐CHA serve as an oxidizing site that can only accept one electron from the metallic Zn, leading to the formation of mononuclear Zn^+^ cations in the zeolites. This was the first report of monoatomic univalent Zn^+^ species in solid materials. However, the applications of Zn^+^‐containing zeolites are still unknown at that stage due to their sensitivity to air and oxygen.

**Figure 4:**
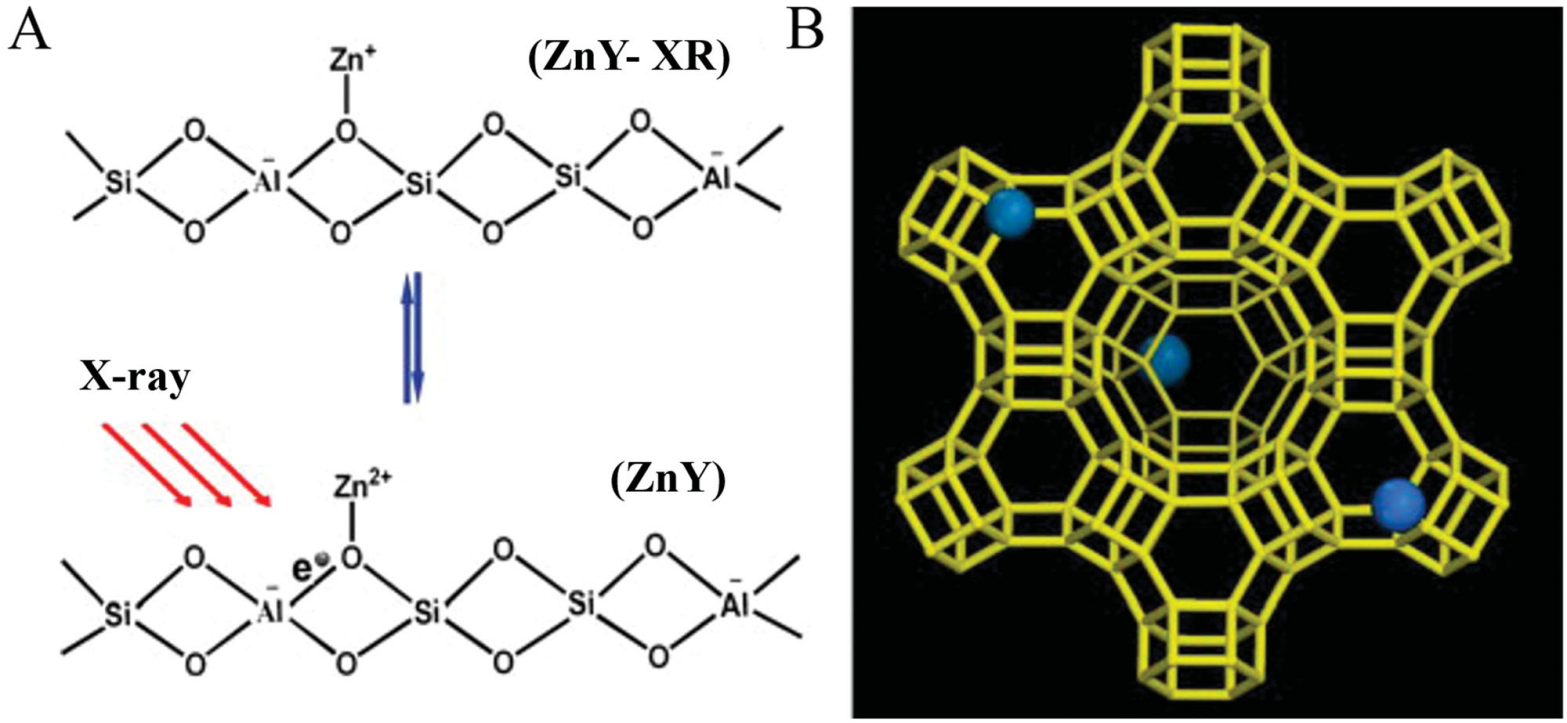
Schematic representations for A) the Zn^+^ species generation from the electron‐rich zeolite Y framework under the X‐ray irradiation, and B) the coordination of the Zn cations in the zeolite Y framework. Reproduced with permission.^34^ Copyright 2013, Elsevier.

Chen's group further developed another approach for the preparation of Zn^+^ species by exchanging Zn^2+^ ions in a ZSM‐5 type zeolite, followed by light excitation.^33^ Briefly, dehydrated HZSM‐5 was reacted with metallic Zn vapor, during which the protons of the Brønsted acidic sites in the zeolite were reduced by Zn atoms to evolve H_2_. The reaction was proposed two involved oxidation reactions: Firstly, a Zn atom reduces two closely positioned protons to form H_2_ and a Zn^2+^ cation, whilst in the other reaction a Zn atom reduces one isolated proton to form Zn^2+^ cation with an extra electron delocalized on the zeolite. Under UV light irradiation, the delocalized electron could get promoted into the Zn 4s orbital, resulting in the formation of Zn^+^ cation species. These as‐formed Zn^2+^ species and Zn^+^ species can mutually interchange with each other under UV light irradiation or thermal treatment. Zn^2+^ sites with an adjacent delocalized electron can also form in dehydrated HY zeolites. Upon X‐ray irradiation, the delocalized electron can be excited from the electron‐rich framework of zeolite Y to the nearby Zn^2+^ cations, thereby generating Zn^+^ species (**Figure** **4**).^34^

**Figure 5:**
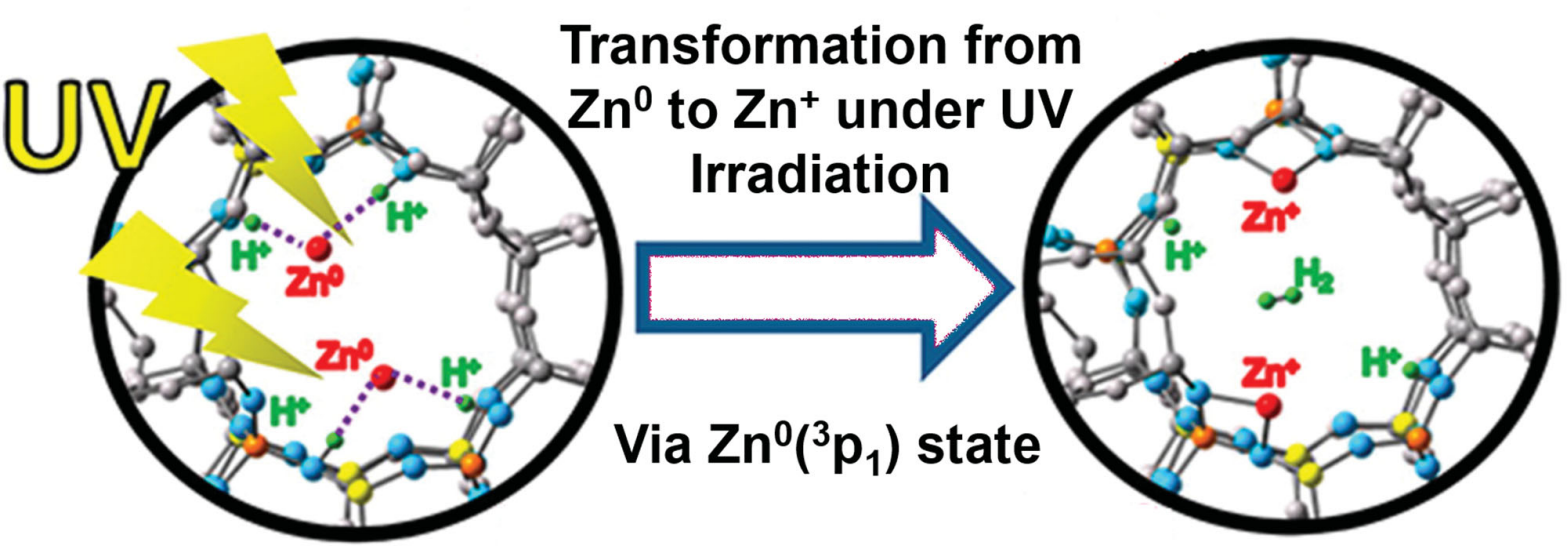
Formation of Zn^+^ species from Zn^0^ encapsulated in zeolite under UV irradiation. Reproduced with permission.^36^ Copyright 2013, American Chemical Society.

In 2012, Kuroda's group discovered an interesting and unprecedented property of Zn^2+^ ions exchanged in zeolites. It was found that Zn^2+^ ions could react with H_2_ to form metallic Zn species via an intermediate zinc hydride species (Equation (1), (2)), Z_A_ and Z_B_ represent the zeolite lattice including Al atoms).^35^ Based on this report, an approach for the synthesis of Zn^+^ species with a paramagnetic nature was developed, involving UV irradiation of Zn encapsulated in the MFI‐type zeolite (**Figure** **5**).^36^ A transformation mechanism was proposed based on UV–vis DR spectra and DFT calculations (see the Characterization section for details), as described in Equation (3). (1)
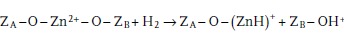

(2)
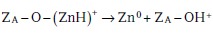

(3)
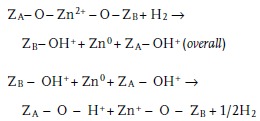



**Figure 6:**
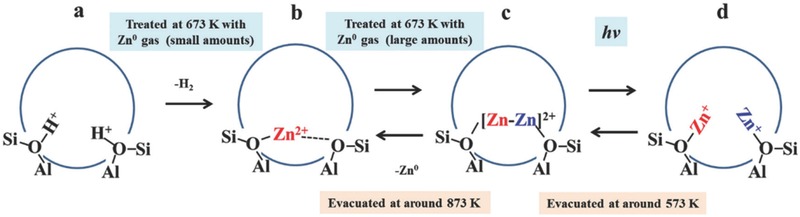
Schematic model for the proposed reaction processes including the formation of the [Zn_2_]^2+^ species caused by the reaction between HMFI and Zn^0^ vapor, as well as the bond dissociation process of the [Zn_2_]^2+^ species. Reproduced with permission.^37^ Copyright 2015, Royal Society of Chemistry.

Post‐synthetic modification of MFI‐type zeolites can also be used to synthesize [Zn_2_]^2+^ and Zn^+^ species, using HMFI and Zn vapor as reactants.^37^ Zn^0^ vapor reacts with HMFI zeolite leading to the initial formation of Zn^2+^‐ion‐exchanged MFI. With increasing exposure to Zn^0^ vapor, [Zn_2_]^2+^ was formed through a spontaneous reverse disproportionation reaction between the Zn^2+^ ions and Zn^0^ vapor. Upon UV‐light irradiation, the [Zn_2_]^2+^ was converted into two stable monomeric Zn^+^ ions through a homogeneous cleavage of the Zn–Zn bond. These two isolated Zn^+^ species could recombine to form the original [Zn_2_]^2+^ species through heating under vacuum (**Figure** **6**). Thanks to the stability of the Si‐O‐Al framework in the MFI zeolite, the Zn^+^ species could also be interconverted with Zn^2+^ under evacuation at higher temperatures, which may show potential for application in photocatalysis due to the high photoactvity of Zn^+^.^33^, ^36^

**Figure 7:**
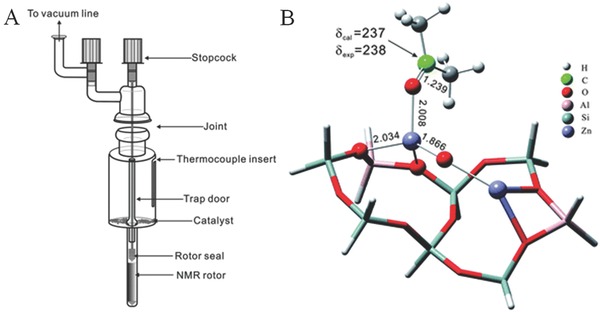
The device for the preparation of ZnZSM‐5 catalyst and the optimized structure of acetone adsorbed on the Zn^+^–O–Zn^2+^ cluster in ZnZSM‐5. Reproduced with permission.^39^ Copyright 2012, Royal Society of Chemistry.

Excited by the special electron transfer feature in zeolite, Deng's group synthesized Zn^+^‐containing ZnZSM‐5 by reacting metallic Zn vapor with HZSM‐5 zeolite in a CAVERN device or a modified glass reactor driven by heat (**Figure** **7**A).^38^, ^39^, ^40^ This synthesis procedure is quite similar to that described by Chen's group, with the CAVERN device convenient for *in situ* NMR analysis. The Zn content determined by inductive coupled plasma (ICP) analysis was 3.8%. Three types of Zn species (isolated Zn^2+^, isolated Zn^+^ and Zn^+^–O–Zn^2+^ clusters) were clearly identified as shown in Figure 7B. Such systems containing isolated Zn cations and Zn^+^–O–Zn^2+^ clusters show great potential in catalysis due to the high electron mobility between these Zn centers.

### 2.3 Synthesis of Zn^+^‐Related Sites in Layered Nanomaterials

**Figure 8:**
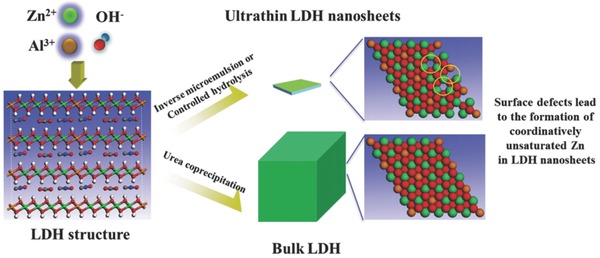
Schematic showing the formation of coordinatively unsaturated Zn cations in ultrathin ZnAl–LDH nanosheets. Reproduced with permission.^49^

The host‐guest nature and microporous structure of zeolites are important to their ability to accommodate Zn^+^ cations. However, the limited number of zeolite hosts limits further development of new Zn^+^‐containing zeolite catalysts. Recently, attention has switched from zeolites to nanomaterials because of their novel electron structures compared to the corresponding bulk materials. During the synthesis of nanomaterials, the surface cations and anions can easily escape from the surface region, leading in some cases to severe structural distortions and high surface defect concentrations as a means of lowering the total surface free energy. Such quantum confinement effects typically generate coordinatively unsaturated metal cations or vacancies, which provide a convenient route for the synthesis of compounds containing metal cations in unusual oxidation states such as Co^3+^, Ni^3+^.^41^, ^42^ Amongst Zn‐incorporated nanoparticles, ZnO with defect structures has been intesively investigated for the catalytic synthsis of methanol and the water shift reation, and oxygen vcancies were proposed to be active sites although Zn^+^ could exsit in those systems and play the real role.^43^ Driess, Cai and co‐workers reported coexisting defects (i.e., interstitial zinc and oxygen vacancies) in defect‐rich nanosized ZnO systems.^44^, ^45^ However, it is yet unclear whether Zn^+^ species exist in such above nanostructured Zn‐containing nanoparticles systems, motivating further investigation. Layered double hydroxides (LDHs) are a class of anion clays consisting of brucite‐like host layers and interlayer anions. The inherent layered structure of LDHs allows control over both nanosheet size and thickness.^46^, ^47^, ^48^ On approaching single nanosheet thicknesses, LDHs offer the potential for generating very high concentrations of coordinatively unsaturated metal cations. Recently, Zhang's group developed a facile strategy for creating coordinatively unsaturated Zn species in ultrathin ZnAl‐LDH nanosheets, by increasing the density of oxygen vacancies (V_o_) during the growth of nanosheets via either an inverse microemulsion technique or controlled hydrolysis (**Figure** **8**).^49^ Advanced characterisation techniques demonstrated that the presence of V_o_ lead to the formation of Zn^+^‐V_o_ complexes in ultrathin LDH nanosheets. More importantly, this facile approach is not limited exclusively to Zn, but is also readily adaptable for generating coordinatively unsaturated metals such as Fe,^50^ Co,^51^ Ni,^14^ Ti,^52^ amongst others.

## 3 Characterization

### 3.1 Electron Spin Resonance

**Figure 9:**
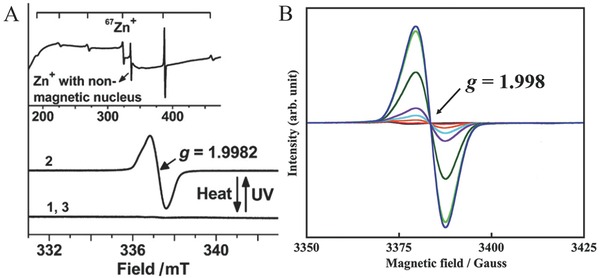
A) Room‐temperature X‐band ESR spectra for Zn^2+^–ZSM‐5 in vacuum (curve 1), the (Zn^+^, Zn^2+^)–ZSM‐5 sample (curve 2), and a reference Zn^2+^–ZSM‐5 sample (curve 3). The inset is the ESR spectrum of the ^67^(Zn^+^, Zn^2+^)–ZSM‐5 sample. B) ESR spectra of the ZnMFI irradiated at various wavelengths. A) Reproduced with permission.^33^ B) Reproduced with permission.^36^ Copyright 2013, American Chemical Society.

Electron spin resonance (ESR) spectroscopy is a powerful technique for detecting paramagnetic Zn^+^ species with a single charged *s*‐electron. Zn^+^ shows a single ESR line with the *g* value of around 1.9980. The Zn^2+^–ZSM‐5 after UV irradiation showed an ESR signal where *g* = 1.9982, indicating the presence of Zn^+^ (**Figure** **9**A). Similarly, Zn^+^ species with *g* value of 1.998 was also observed after UV excitation of Zn^0^ species encapsulated in a MFI‐type zeolite, with the intensity of the ESR signal being dependent on the excitation wavelength (Figure 9B).^36^ In further experiments using the ^67^Zn isotope, the hyperfine interaction provided further evidence for the existence of Zn^+^ species. Popescu and co‐workers firstly observed a large hyperfine splitting and the high isotropy of *g* and hyperfine interaction tensors (*g*_||_ = 2.0008 ± 0.0002; *g_⊥_* = 1.9965 ± 0.0002; *A*_||_ = 515.5 ± 0.2 G; *A*_⊥_ = 505.3 ± 0.2 G) for ^67^Zn^+^ in calcite,^53^ which is consistent with the (4s)^1^ electron configuration of Zn^+^. Similarly, the presence of Zn^+^ cations excited by UV‐light irradiation in (Zn^+^, Zn^2+^)–ZSM‐5 was confirmed by reacting HZSM‐5^−^ with a ^67^Zn‐enriched source (97%). After irradiation, the ESR spectrum of the ^67^Zn^2+^–ZSM‐5^–^ sample exhibited six hyperfine lines because of the interaction of the unpaired 4s electron of ^67^Zn^+^ with the I = 5/2 nuclear spin (inset of Figure 9A).^33^ These studies demonstrate that ESR is a powerful technique for detecting paramagnetic Zn^+^ cations, and the six hyperfine lines of ESR clearly gave the evidence for the formation of Zn^+^ species. However, due to the similar signal position of paramagnetic unpaired electrons in Zn^+^ and other metal cations like Ti^3+^ with *g* values around 1.99, along with the influence of surrounding environment, it is preferable to use ESR in parallel with other spectroscopic techniques to unambiguously identify Zn^+^ cations.

### 3.2 X‐ray Structure Analysis

**Figure 10:**
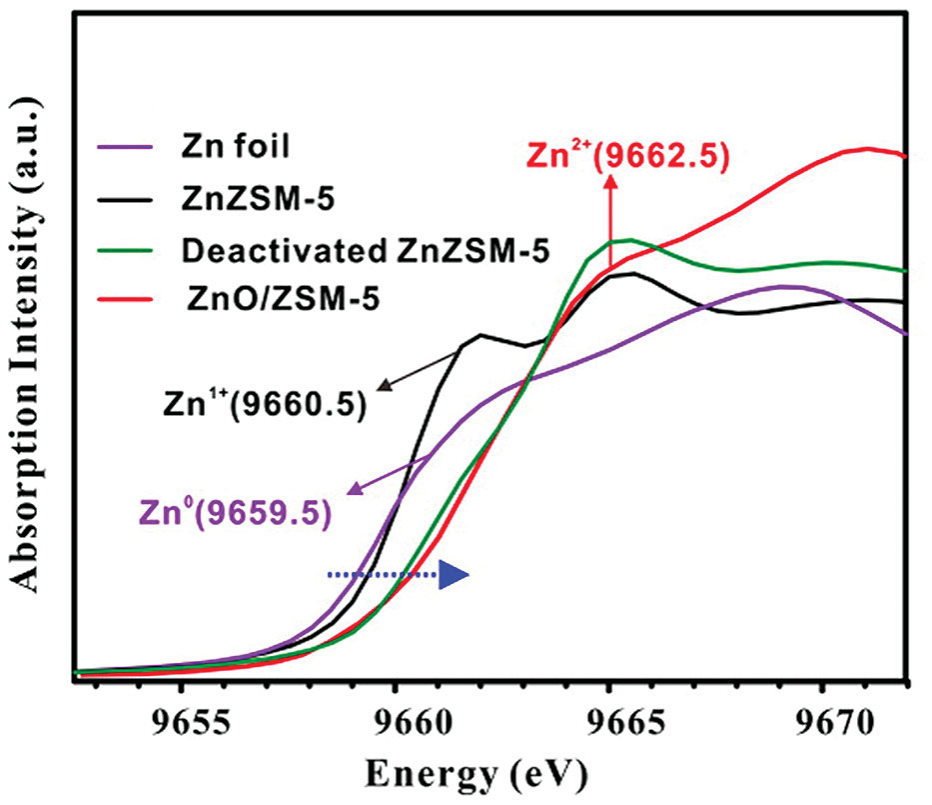
Zn K‐edge XANES spectra of fresh ZnZSM‐5, deactivated ZnZSM‐5, Zn foil and ZnO/ZSM‐5 reference materials. Reproduced with permission.^40^ Copyright 2013, American Chemical Society.

For the organometallic compounds described in section 2.1, X‐ray single‐crystal diffraction analysis provided information about their molecular structure. Results demonstrated that decamethyldizincocene and its corresponding derivatives, linear tri‐zinc complex [LZnZnZnL] (L = bulky amide), and multicenter‐bonded polyzinc compounds with an unusual cubic [Zn_8_(HL)_4_(L)_8_] core contain a Zn–Zn bond (Figure 2), suggesting the presence of Zn^+^ species. X‐ray absorption fine structure (XAFS) spectroscopy is a particularly useful tool to study the local structure of a specific atom to explore the valence state, vacant orbitals, electronic configuration, interatomic distance, coordination number and coordination geometry.^54^, ^55^ XAFS can be divided into two parts, X‐ray absorption near edge structure (XANES) and extended X‐ray absorption fine structure (EXAFS). As a general rule, metal absorption edges shift to higher photon energies with increasing oxidation state. For Zn‐containing sieve systems, the Zn K‐edge absorption edge of freshly prepared ZnZSM‐5 falls intermediate between those of metallic Zn (Zn^0^) and ZnO/ZSM‐5 (Zn^2+^) reference materials, implying that the Zn^+^ ions coexist with the dominant Zn^2+^ ions in the ZnZSM‐5 sample (**Figure** **10**).^40^

**Figure 11:**
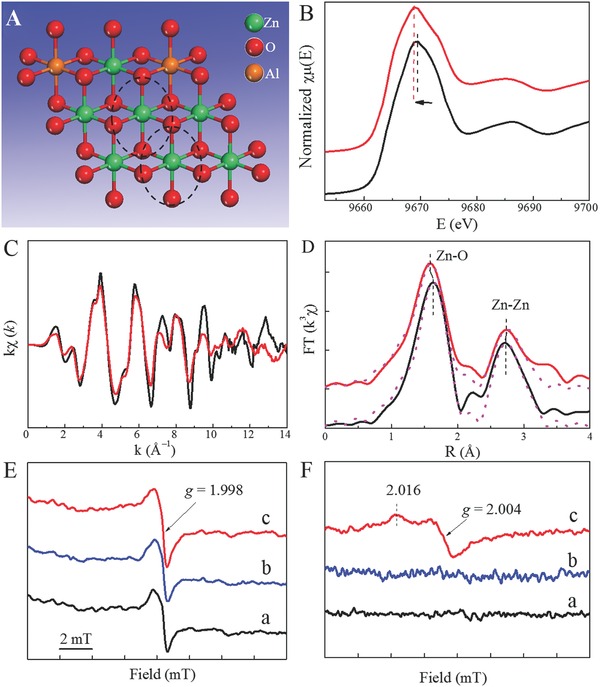
A) Schematic structure model of a ZnAl–LDH single layer. B) Zn K‐edge XANES spectra, C) Zn K‐edge extended XANES oscillation functions *k* ^2^ *χ* (k), and D) magnitude of *k* ^2^‐weighted FT of Zn K‐edge EXAFS spectra for ZnAl‐nanosheet (red) and the counterpart ZnAl‐bulk (black). ESR spectra of E) ZnAl–LDH nanosheets and F) ZnAl–LDH bulk under different irradiation. Reproduced with permission.^49^

Similarly, XAFS analyses by Zhang's group^49^ confirmed the existence of coordinatively unsaturated Zn ions in ultrathin ZnAl‐LDH nanosheets. **Figure** **11**A shows the atomic structure model of a typical LDH layer. The Zn K‐edge spectral peak maximum for ZnAl‐LDH nanosheets is lower than that of ZnAl‐LDH‐bulk (Figure 11B), whilst the Zn K‐edge EXAFS oscillation was reduced for ZnAl‐LDH nanosheets compared to ZnAl‐LDH‐bulk (Figure 11C). Results suggest that ZnAl‐LDH nanosheets contain Zn in both a lower average oxidation state (Zn*^d^* ^+^, 1 < *d <* 2) and lower average coordination number than in ZnAl‐LDH‐bulk. From the Zn K‐edge EXAFS analysis (Figure 11D and **Table** **1**), it is evident that the Fourier transform of the Zn EXAFS for the ZnAl‐LDH nanosheets is weaker and the detailed structure of the first Zn–O shell changed dramatically compared to the bulk compound, with a shorter Zn–O distance (2.06Å), as well as a lower Zn coordination number (5.9). The data reflect a severe structural distortion about Zn^+^ centers in these ultrathin nanosheets. The XAFS data complement ESR spectra collected for the ZnAl–LDH nanosheets, where a peak with a *g* value around 1.998 was attributed to the formation of Zn^+^–V_o_ complexes (Figure 11E,F).

**Table 1:** Local structure parameters around Zn estimated by EXAFS analysis

Samples	Shell	Na)	*R* b) [Å]	*σ* ^2^ [Å^2^]	Δ*E*_0_ [eV]
Zn(OH)_2_	Zn–O	4.0	1.97	0.0040	2.4
	Zn–Zn	12.0	3.22	0.0098	2.8
ZnAl–LDH bulk	Zn–O	6.0	2.08	0.0080	2.0
	Zn–Zn	4.0	3.10	0.0090	–1.6
ZnAl–LDH nanosheets	Zn–O	5.9	2.06	0.0098	0.1
	Zn–Zn	3.6	3.13	0.0112	–1.2

^a)^ *N* = coordination number;

^b)^ *R* = distance between Zn and O atoms. Data from Supporting Information of Ref. 49.

### 3.3 DFT Calculations

Over the past decade, DFT calculations have emerged as a particularly useful tool for modeling the electronic structure and electron transformations of inorganic compounds, and have greatly improved understanding of Zn^+^‐containing species.^56^ DFT calculations provide detailed information about the occupancy state of the orbitals, chemical bonding structure as well as defect types, their location and concentration, all of which are difficult to determine by experimental studies. DFT calculations are also being used increasingly to establish structure‐activity relationships in catalytic systems.

**Figure 12:**
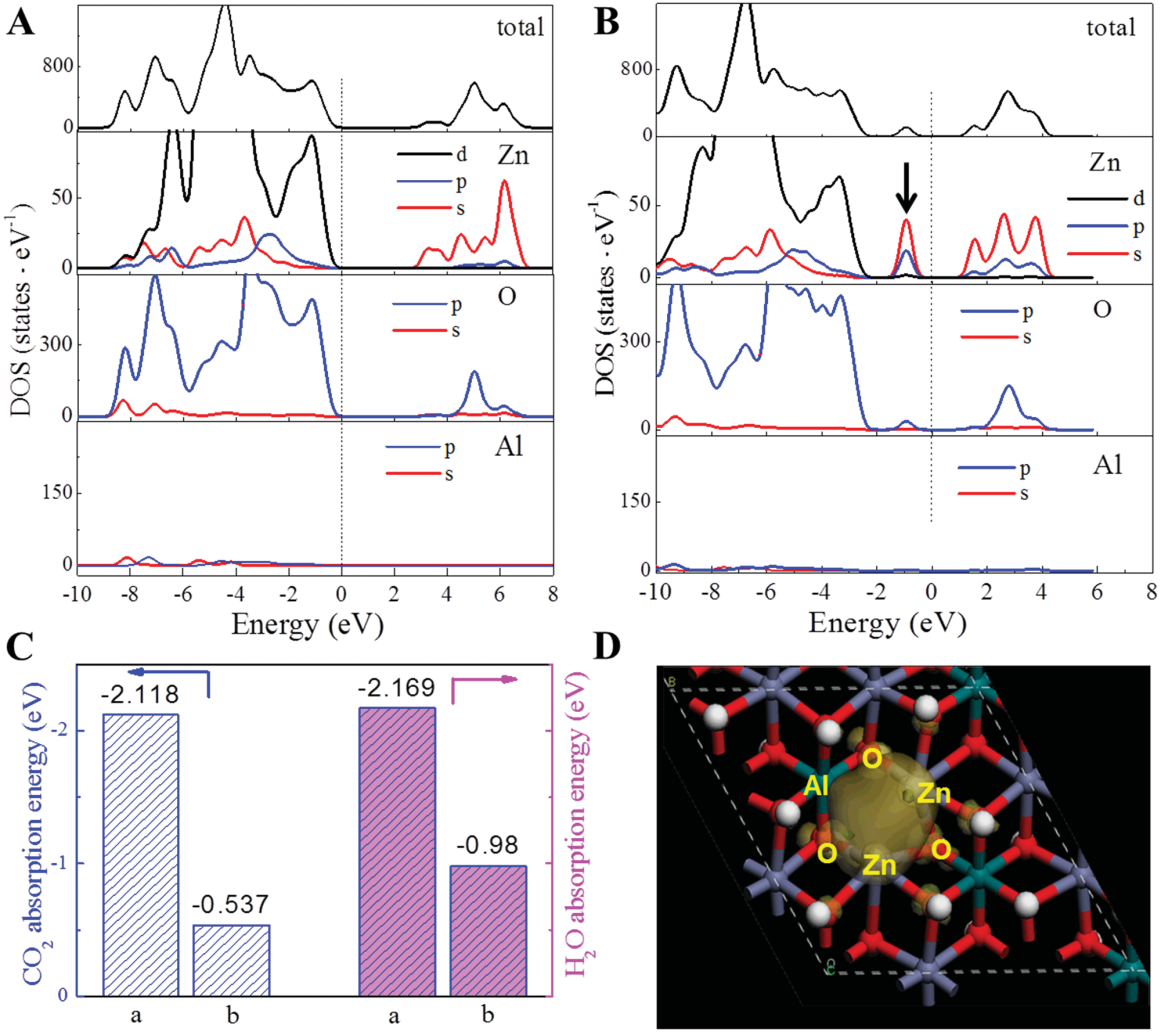
Total and partial electronic density of states for: A) bulk ZnAl–LDH and B) V_o_‐doped ZnAl–LDH system. C) DFT‐calculated adsorption energies of CO_2_ and H_2_O molecules on LDH with or without defects. D) Charge‐density distribution of the defective ZnAl‐LDH. Reproduced with permission.^49^

The electronic structure of the Zn‐containing LDHs with or without surface defects was studied recently.^49^ ZnAl–LDH with surface defects was modeled by removing OH groups adjacent to the Zn centers, thereby creating a V_o_ and a coordinatively unsaturated Zn defect. **Figure** **12**A shows that for the defect‐free Zn–LDH system, the electrons can be excited from the top of the valence band composing occupied Zn 3d and O 2p orbitals to the unoccupied Zn 4s orbitals, consistent with Zn^2+^ with no electron in a 4s orbital. For ZnAl–LDH with oxygen defects (Figure 12B), a new defect level appeared in the band gap. The defect energy level results from hybridization of occupied Zn 4s orbitals and O 2p orbitals to give a covalent bond. The appearance of this defect level confirms the existence of a single Zn 4s orbital electron, consistent with a Zn^+^–V_o_ complex as suggested experimentally by ESR and EXAFS. Furthermore, the Zn^+^–V_o_ complex‐doped LDH possesses an improved adsorption energy for CO_2_ and H_2_O compared to that of defect‐free LDH with a high apparent charge density around Zn^+^–V_o_ sites (Figure 12C,D). These results indicate that the adsorption of reactants by defective LDH is more favorable, enhancing catalytic activity towards CO_2_ photoreduction.

**Figure 13:**
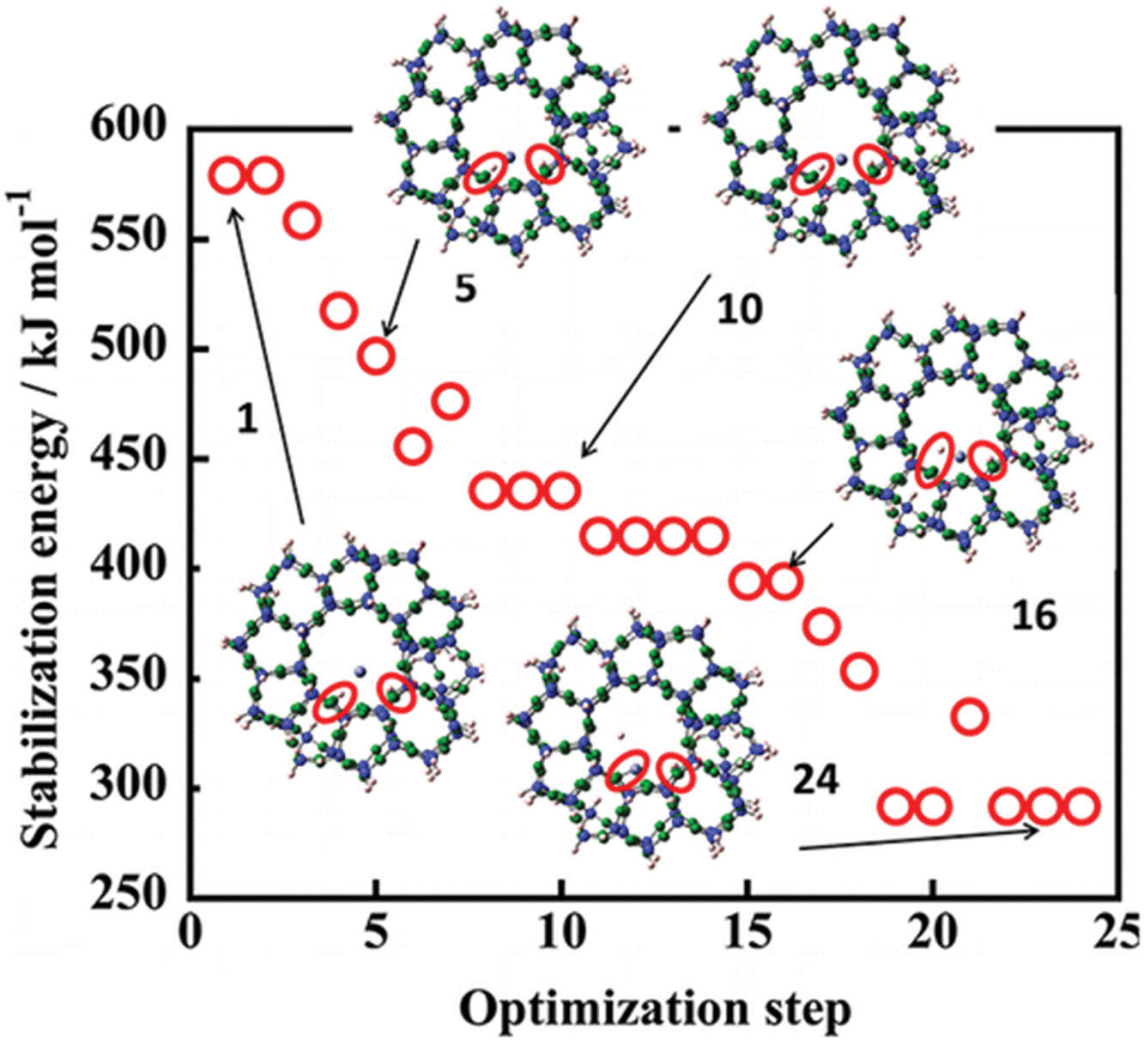
Energy relationship in the changing process from Zn^0^ to Zn^+^ by way of the ^3^P state of an atomic zinc formed in MFI. Reproduced with permission.^36^ Copyright 2013, American Chemical Society.

DFT calculations have also been used by other groups to explain the formation mechanism of Zn^+^ species. Kuroda's group used DFT calculations to identify the formation mechanism and the final structure of Zn^+^ in a MFI zeolite under UV irradiation.^36^ Excitation of the 4s–4p transition of an atomic Zn^0^ species grafted in MFI by UV light created an excited singlet state (^1^P), which in turn generated an excited triplet state (^3^P) via intersystem crossing (**Figure** **13**). This ^3^P state leads to the formation of Zn^+^ and H^•^ (radical) and the final structure [Zn^+^–(H^+^)MFI + H^•^], where the Zn^+^ ion was positioned primarily on the M7 site through interaction with the two oxygen atoms near the substituted Al atoms. The H^•^ species was floating in the MFI pore (Figure 5, right).

### 3.4 UV–Vis DR Spectroscopy

**Figure 14:**
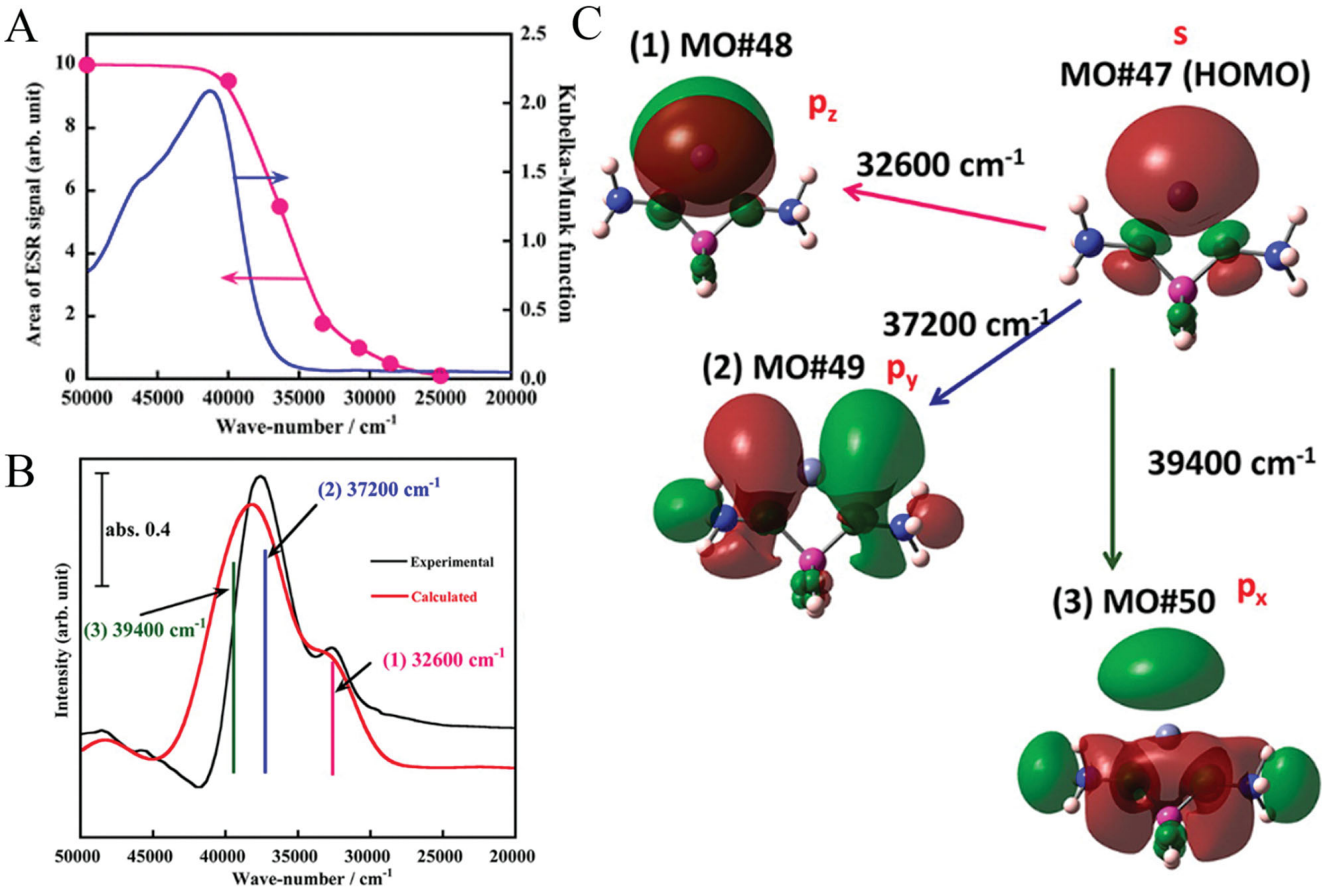
A) Band areas of ESR signal corresponding to the respective spectra shown in Figure 9B, together with the absorption spectrum of Zn^0^(H^+^)_2_MFI‐95. B) Experimentally (black line) and theoretically (red line) UV–vis DR spectra of Zn^I^ MFI. C) Respective components (from 1 to 3) obtained by the DFT calculations. Reproduced with permission.^36^ Copyright 2013, American Chemical Society.

UV–vis DR spectroscopy can also be used to study the formation of Zn^+^ species. Kuroda's group reported that Zn^0^ atoms encapsulated in a zeolite host showed strong absorption bands in the region between 50,000 and 33,000 cm^–1^ (**Figure** **14**A**,** blue line), which is readily explained by considering the electronic 4s–4p transition of Zn^0^ species.^35^ The appearance of bands at around 38 000 and 32 500 cm^–1^, together with a decrease in intensity around 42 000 cm^–1^ (Figure 14B, black line) under appropriate light excitation can be used as confirmation for the formation of Zn^+^. From the wavelength‐dependent UV–vis DR spectra, ESR absorption spectrum band areas (Figure 14A) and DFT calculations (Figure 14B, red line, and Figure 14C), can all be used to document the transformation of Zn^0^ to Zn^+^ species.^36^

## 4 Applications

### 4.1 Methane Conversion

Conversion of methane to transportation fuels and chemical feedstocks represents a hot area of current research in the field of catalysis.^57^ For several decades, considerable effort has been devoted to the methane activation.^58^, ^59^, ^60^, ^61^, ^62^ The process is challenging because methane possesses a high C–H bond energy (434 kJ mol^–1^), negligible electron affinity, large ionization energy as well as low polarizability.

**Figure 15:**
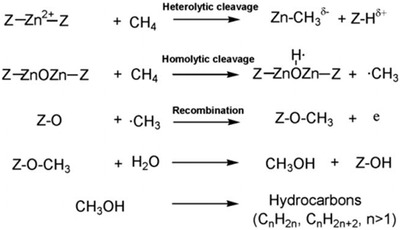
Methane activation and subsequent conversion pathways on ZnZSM‐5 catalyst (Z donates zeolite support). Reproduced with permission.^39^ Copyright 2012, The Royal Society of Chemistry.

Conversion of methane into higher hydrocarbons via a methanol‐to‐gasoline (MTL) process is a general and effective approach for methane conversion.^63^ The ZnZSM‐5 catalyst with three types of Zn species (isolated Zn^2+^, isolated Zn^+^ and Zn^+^–O–Zn^2+^ clusters) described above shows excellent catalytic performance for methane conversion to higher hydrocarbons at room temperature in the presence of water.^39^ *In situ* solid‐state NMR spectroscopy and theoretical calculations suggest the conversion process goes through the following steps: The isolated Zn^2+^ served as the active site for the formation of zinc methyl species via heterolytic dissociation of C–H bond. The Zn^+^–O–Zn^2+^ site is responsible for the formation of surface methyl radicals via hemolytic cleavage of a C–H bond, which subsequently react with an oxygen atom of the zeolite support to give methoxy species. Methoxy species are important intermediates for the formation of methanol and further conversion to higher hydrocarbons through an MTL process (**Figure** **15**). The isolated Zn^+^ in the ZnZSM‐5 catalyst is a spectator in the conversion of methane, as evidenced by DFT calculations.

**Figure 16:**
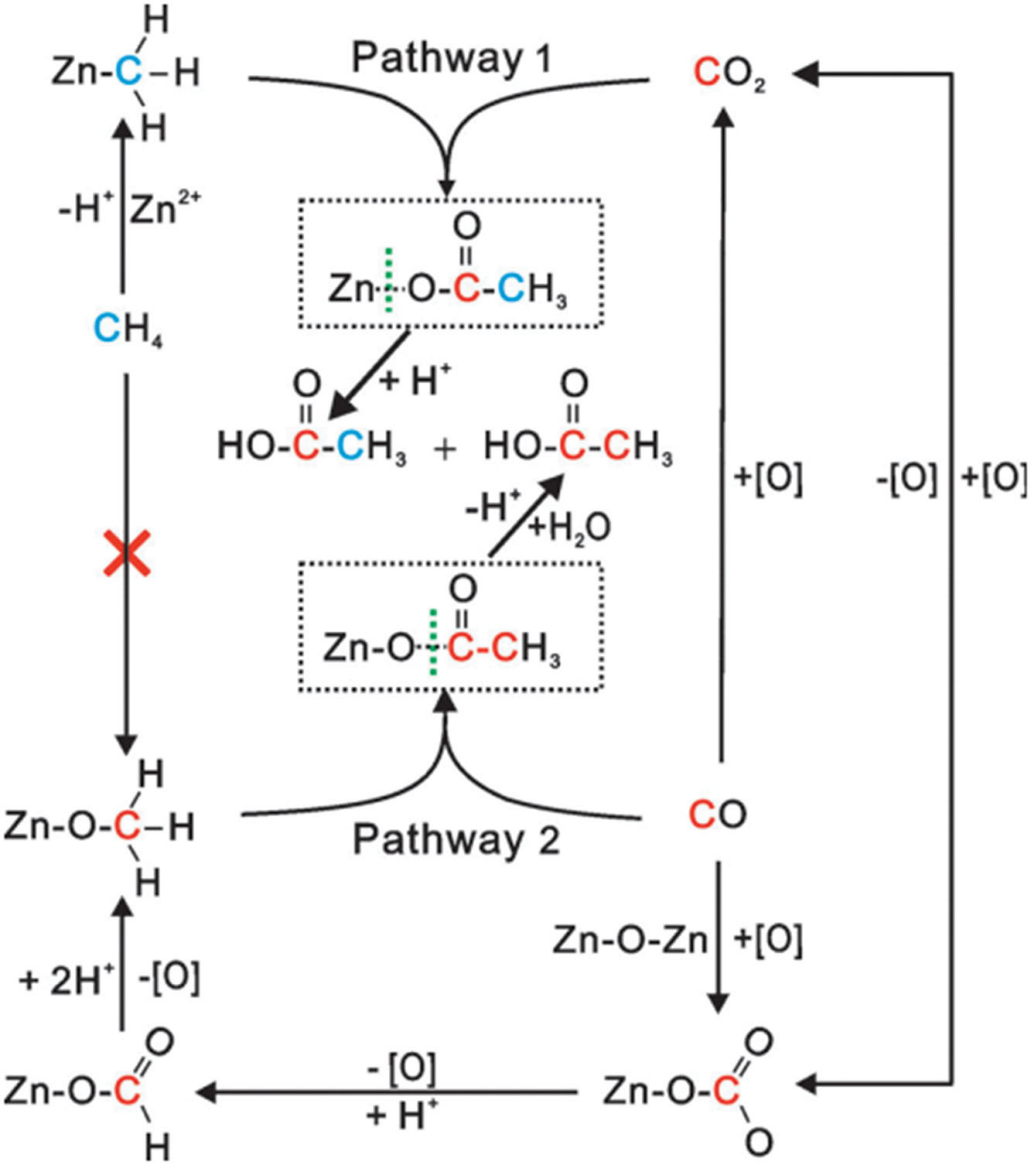
Scheme of proposed reaction pathways for the formation of acetic acid from methane and CO on ZnZSM‐5 catalyst. Reproduced with permission.^38^

The multi‐step MTL process requires high temperatures, which makes its challenge for the large scale production of fuels.^64^ Direct carbonylation of methane with CO to form higher hydrocarbons, such as acetic acid, is considered to be a more economically viable and environmental‐friendly approach for methane transformation.^65^ The ZnZSM‐5 catalyst also shows good catalytic performance for the direct carbonylation of methane into acetic acid with CO under mild conditions (573–623 K).^38^ The carbonylation process was monitored using ^13^C isotope labeled *in situ* solid‐state NMR spectroscopy.^31^ Two competitive parallel pathways were identified for the formation of acetic acid (**Figure** **16**). In the first pathway, CO is activated to form methoxy intermediates through an oxidation and hydrogenation process, which can further react with CO to generate acetic acid through a Koch‐type mechanism (Pathway 1). Simultaneously, methane is activated to give zinc methyl intermediates that can react with CO_2_ through a typical organometallic reaction to yield acetic acid (Pathway 2). Zn^+^–O–Zn^2+^ clusters are the probable oxidation centers, with the H^+^ arising from either the bridging hydroxy groups of the ZnZSM‐5 catalyst or from the heterolytic dissociation of methane. These findings demonstrate the potential of Zn‐modified zeolite catalysts for the selective conversion of small alkanes and co‐reactants into more valuable chemicals.

**Figure 17:**
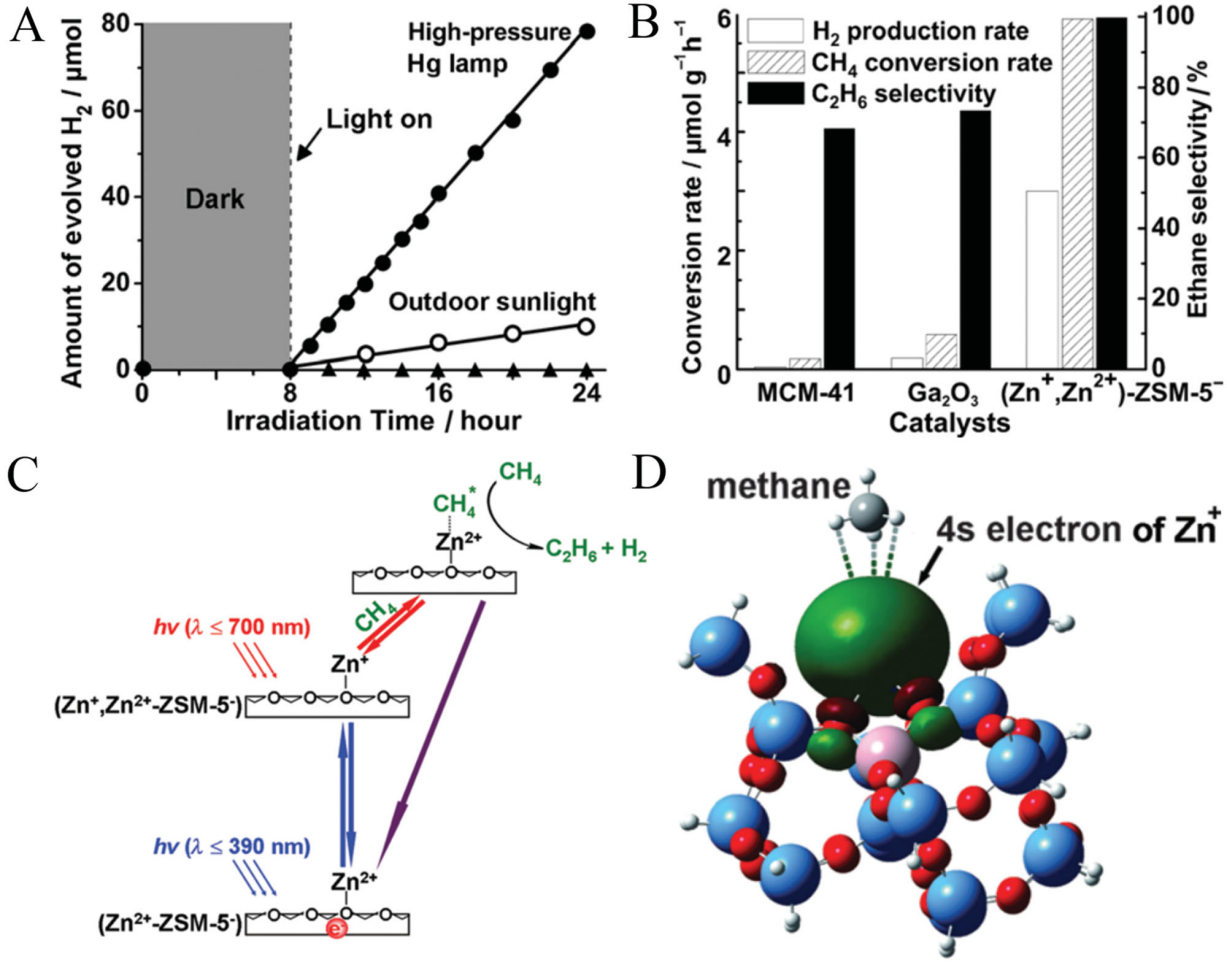
A) Photocatalytic hydrogen evolution of NOCM reaction catalyzed by (Zn^+^, Zn^2+^)–ZSM‐5^–^ catalyst. B) Methane and hydrogen production rate, and ethane selectivity obtained for the NOCM reaction catalyzed by different photocatalysts. C) Schematic energy diagram for the processes of the photocatalytic reaction. D) The optimized geometry of methane attracted by the Zn^+^ active site. Reproduced with permission.^33^

The direct non‐oxidative coupling of methane (NOCM) is another promising approach for methane conversion, which is considered to be economic and environmentally friendly.^66^, ^67^, ^68^ The (Zn^+^, Zn^2+^)–ZSM‐5^–^ catalyst synthesized by Chen's group showed excellent catalytic activity and superior selectivity for the NOCM to ethane under UV or direct solar irradiation, compared to traditional NOCM catalysts (**Figure** **17**A,B). A catalyst with small pores (diameter 0.55 nm) showed excellent selectivity for the formation of ethane (>99.6%) compared to a large pore modified zeolite and superior photocatalytic performance compared to Zn^+^ modified zeolites with a high Si/Al ratio. These results indicate that the framework structure of the zeolites plays a crucial role in the product selectivity, possibility a consequence of the special pores structure. In addition, the light response of Zn^+^, Zn^2+^ species is not the same. (Zn^+^, Zn^2+^)–ZSM‐5^−^ photocatalysts for NOCM lose activity gradually upon visible light irradiation, with the ESR signal of Zn^+^ disappearing after 8 h in the presence of methane. This is ascribed to the 4s electron of Zn^+^ being transferred back to the zeolite framework during the interaction with methane. Under UV irradiation, the ESR‐silent catalyst retained its photocatalytic activity, because the single electron transfering from the zeolite framework to the 4s orbital of the Zn^2+^ cation is driven by UV light (Figure 17C). Meanwhile, the DFT calculations (Figure 17D) demonstrate the methane adsorption sites and the mechanism of electron transfer on the active Zn^+^ sites. This is the first example of direct methane conversion driven by light irradiation, and this novel idea has since been expanded to other systems such as Ga^3+^‐modified ETS‐10 for the photoactivation of the C–H bond of methane.^69^ This work provides valuable new insight into the C‐H bond activation using Zn^+^‐containing heterogeneous catalyst at room temperature, and inspires the design of other novel photocatalysts for the NOCM reaction.

### 4.2 Alkylation of Benzene

**Figure 18:**
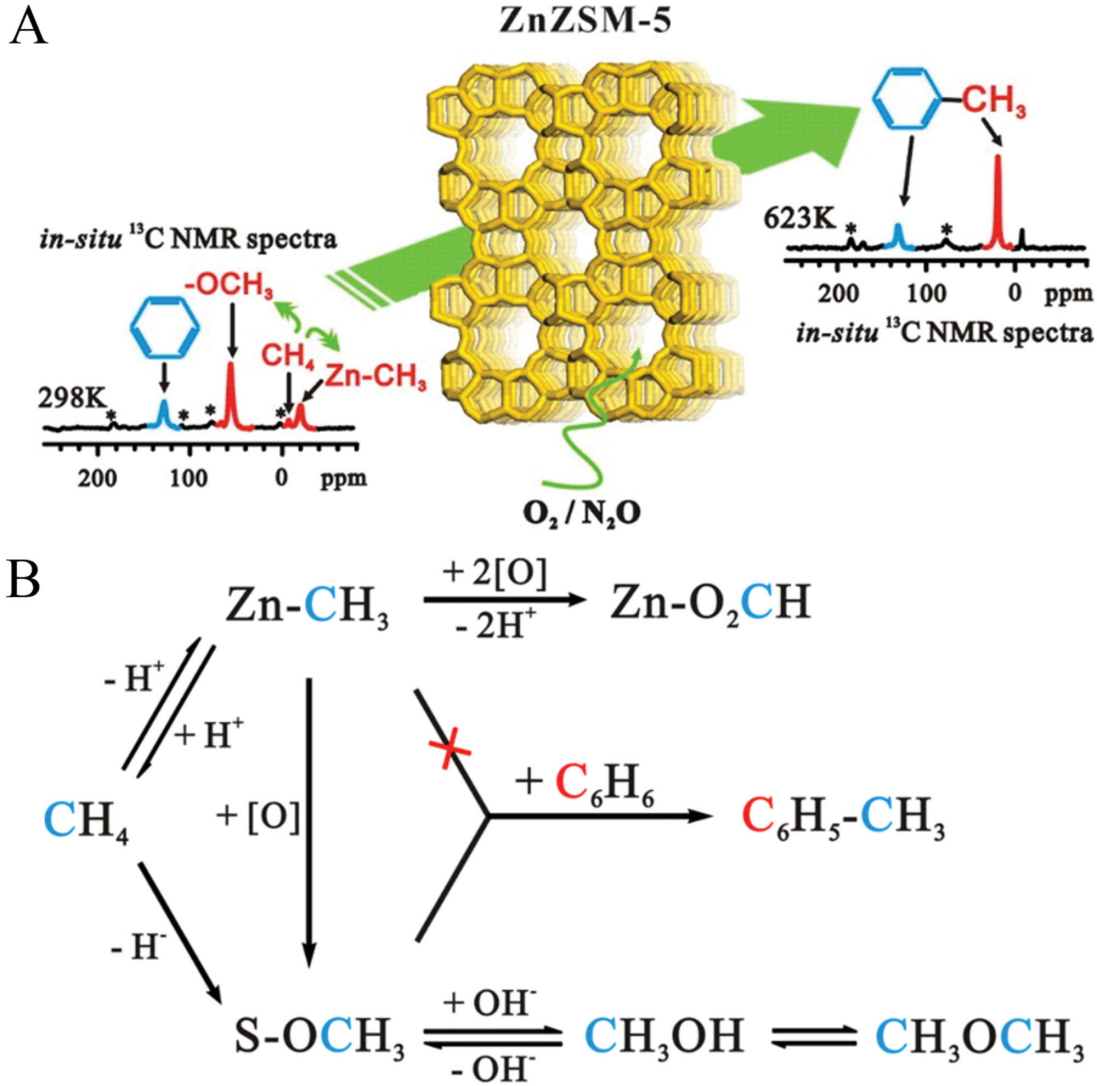
Scheme presentation of A) alkylation of benzene with methane on ZnZSM‐5 catalyst. B) Proposed reaction pathway for the formation of toluene from methane and benzene on ZnZSM‐5 catalyst in an oxidizing atmosphere (S denotes zeolite support). Reproduced with permission.^70^ Copyright 2013, American Chemical Society.

In addition to the activation of methane, ZnZSM‐5 catalysts also show good catalytic ability for the alkylation of benzene with methane using O_2_ or N_2_O as the oxidant at temperatures between 523–623 K (**Figure** **18**A).^70^ *In situ* solid‐state NMR spectroscopy,^13^C isotope labeled reactants, and GC‐MS analysis were used to explore the reaction process. In this reaction, methane was activated into methoxy species and zinc methyl intermediates on ZnZSM‐5. The positively charged methyl group of the methoxy species serves as an electrophile, directly interacting with benzene to produce toluene. Zinc methyl species are indirectly involved in the methylation of benzene, and can be oxidized into methoxy species (Figure 18B). This study reveals an exciting pathway for methane conversion into more valuable chemicals under mild conditions. However, the stability and the cycling of Zn‐modified zeolite catalysts need to be explored further.

**Figure 19:**
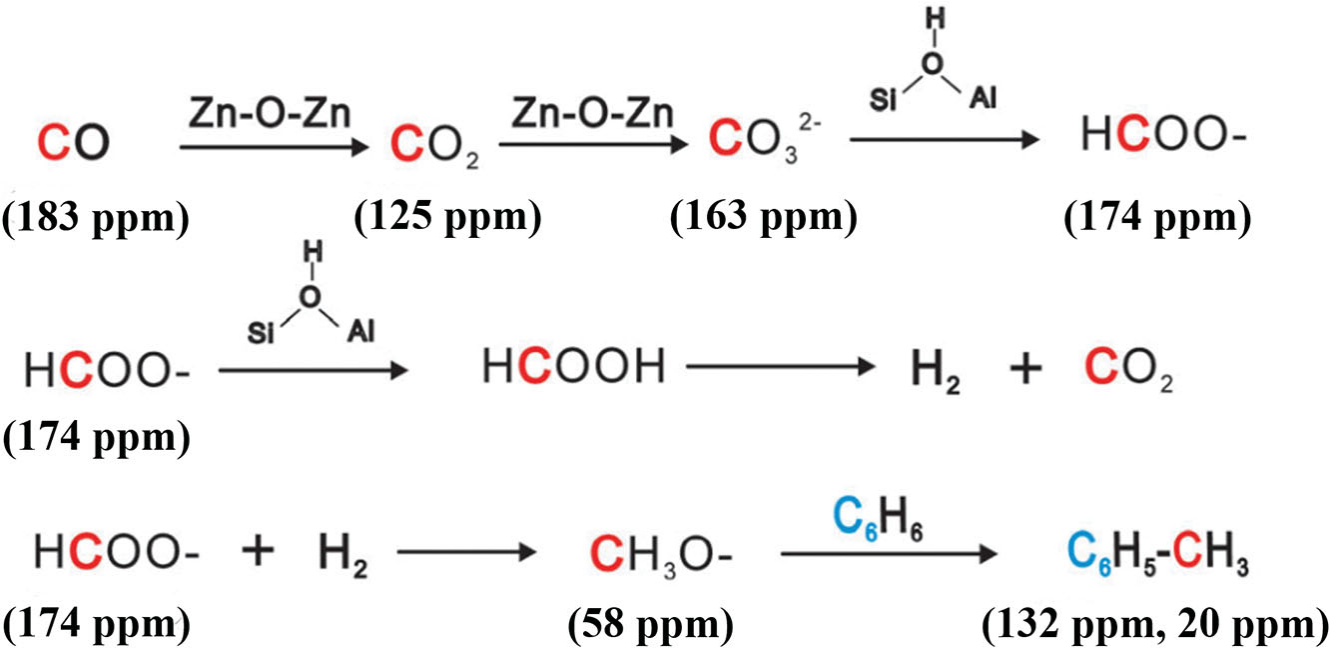
Scheme of proposed reaction pathway for the formation of toluene from alkylation of benzene with CO over the ZnZSM‐5 catalyst. Reproduced with permission.^71^ Copyright 2014, Royal Society of Chemistry.

The alkylation of benzene can also be driven with CO over a ZnZSM‐5 catalyst.^71^ During the process, CO is firstly oxidized into CO_2_ on the Zn^+^–O–Zn^2+^ clusters in ZnZSM‐5, and then CO_2_ is transformed into carbonate species. The carbonate species are then converted into formate species through hydrogenation by proton transfer from the Bronsted acid site of the HZSM‐5 support. The subsequent hydrogenation of the formate species yields methoxy species which react with benzene to produce toluene (**Figure** **19**). The bifunctional nature of the ZnZSM‐5 catalyst provides a viable way to activate CO and promote the alkylation reaction.

The above works successfully demonstrate the potential of Zn‐modified zeolite catalysts for methane activation and conversion into more valuable chemicals under mild conditions. We expect more exciting results about methane conversion would be reported in the near future.

### 4.3 CO Oxidation at Low‐Temperature

**Figure 20:**
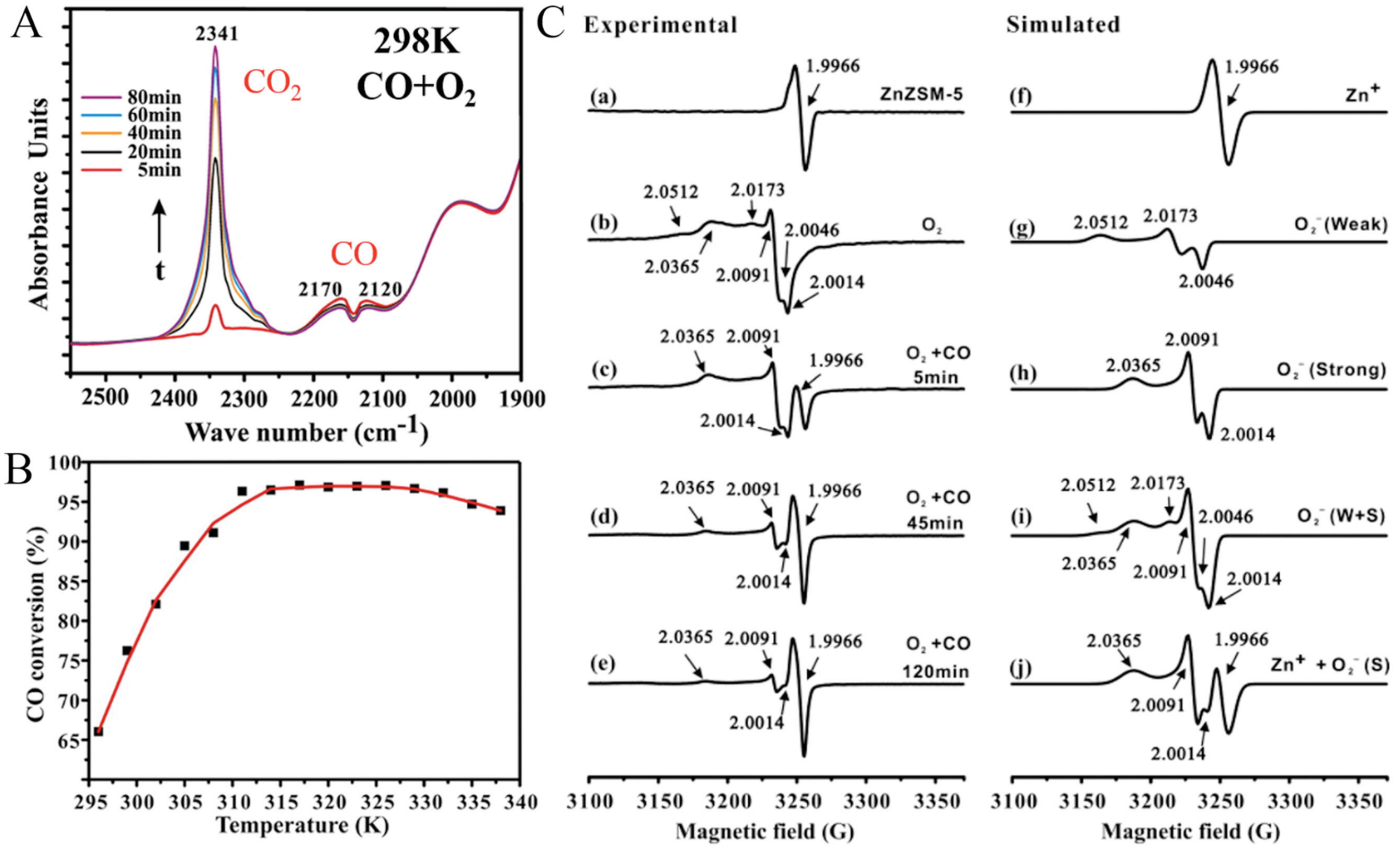
A) *In situ* DRIFT spectra of CO oxidation on ZnZSM‐5 at 298 K. B) CO conversion at different temperatures. C) *In situ* ESR spectra of ZnZSM‐5 recorded at 298 K. Reproduced with permission.^40^ Copyright 2013, American Chemical Society.

Low‐temperature oxidation of CO has practical importance in many industrial processes.^15^ Noble‐metal supported catalysts showed excellent low temperature activity for CO oxidation, but the high price of noble metals and their limited abundance restrict their applications. Interestingly, monovalent Zn^+^ confined in the ZSM‐5 zeolite catalyst exhibited very good catalytic performance for low‐temperature CO oxidation with a conversion of 65% at room temperature (**Figure** **20**A,B). *In situ* diffuse‐reflectance infrared Fourier transform (DRIFT) and ESR spectroscopy experiments clarified the mechanism for CO oxidation, wherein Zn^+^ serves as active site. O_2_ is activated by Zn^+^ ions at room temperature, leading to the formation of Zn^2+^ and active superoxide (O_2_^–^) species. Subsequently, the O_2_^–^ species oxidize CO to CO_2_, with the Zn^2+^ being reduced back to Zn^+^. The electron transfer between molecular O_2_ and Zn^+^ represent a perfect catalytic cycle for CO oxidation (Figure 20C). This work demonstrates that Zn‐containing zeolite catalysts represent viable alternatives to the widely used Fe, Co and noble metal (Au, Pt) catalysts for the low temperature oxidation of CO.

### 4.4 CO_2_ Photoreduction

Carbon dioxide emissions caused by fossil fuel combustion for electricity generation and transportation contribute directly to global warming. Various approaches have been proposed to address CO_2_ emissions, including CO_2_ capture and storage,^72^ electrocatalytic reduction^73^, ^74^ or photocatalytic reduction^75^, ^76^ of CO_2_. The latter two processes are particularly attractive as they can yield valuable chemicals or fuels. Photoreduction of CO_2_ to valuable chemicals using clean and renewable solar energy is considered to be the most desirable approach for reducing CO_2_ emissions and addressing growing global demand for transportation fuels.

**Figure 21:**
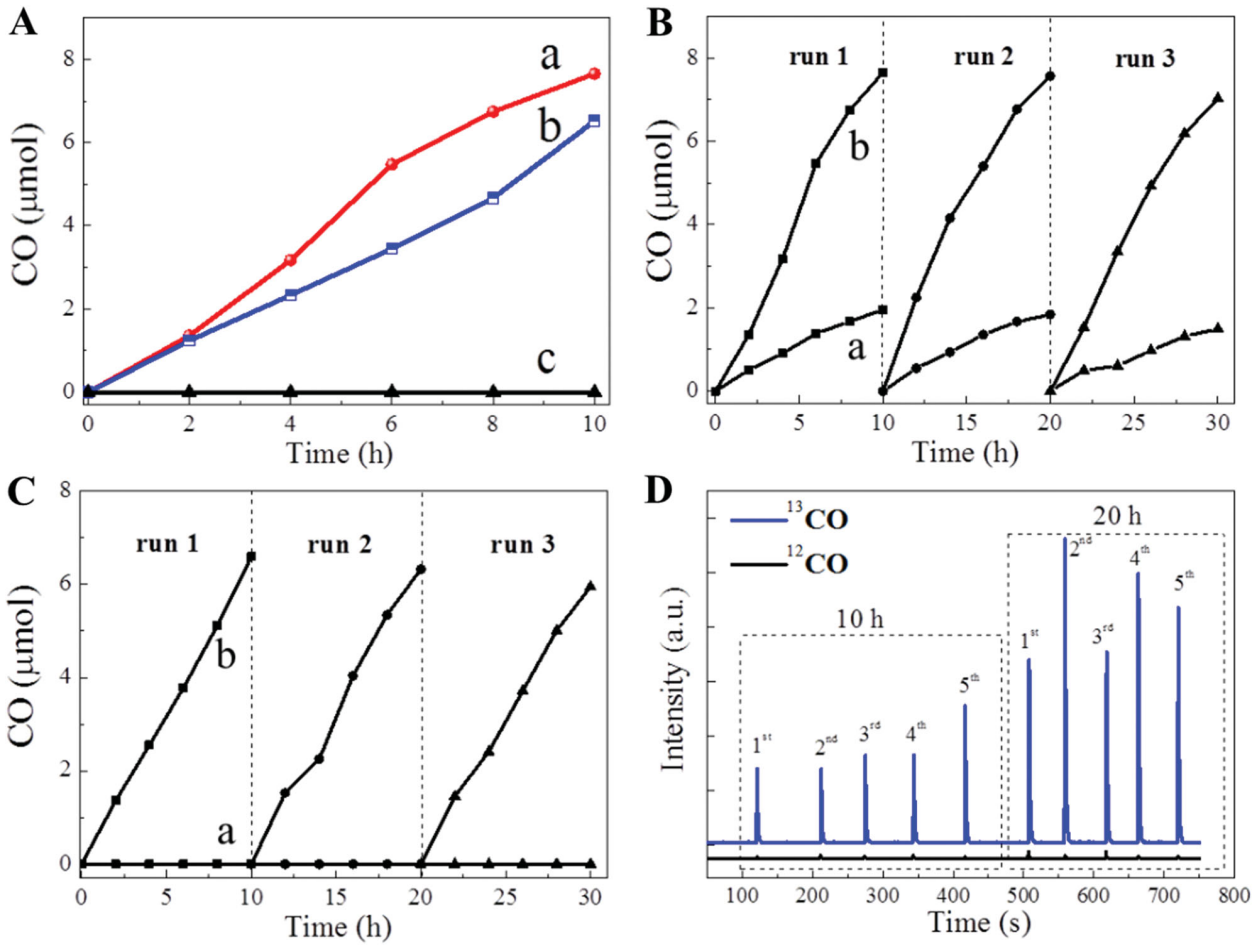
A) Time course of CO evolution in the photoreduction of CO_2_ with water under UV–vis light: a) ZnAl‐LDH nanosheets with size of 30 nm, b) ZnAl‐LDH nanosheets with size of 70 nm, and c) ZnAl–LDH bulk. Catalyst cycling studies of (B) and (C) ZnAl–LDH nanosheets with different particle size under a) Ar atmosphere and b) CO_2_ with water vapor. D) Isotope labelled ^13^CO_2_ experiments by ZnAl–LDH nanosheets. Reproduced with permission.^49^

Recently, the ultrathin ZnAl‐LDH nanosheets (platelet size of 40 nm with 2 repeat stacking layers) with coordinatively unsaturated Zn ions (Zn^+^–V_o_ complex) showed excellent activity for the photoreduction of CO_2_ to CO with water under UV–vis light irradiation. The catalytic activity was ≈20 times higher than that of a commercial ZnO nanoparticle reference photocatalyst under the same reaction conditions (**Figure** **21**A). The coordinatively unsaturated Zn‐containing ZnAl–LDH nanosheets also showed excellent stability up to at least 30 h (Figure 21B,C). Experiments using isotopically labelled ^13^CO_2_ demonstrated that the product CO originated from the initial raw reactant CO_2_ (Figure 21D). Detail characterization studies revealed that the coordinatively unsaturated Zn centers in the LDH serve as trapping sites to promote the adsorption of CO_2_ (Figure 12C) and facilitate electron transfer to the reactant, thereby enhancing photocatalytic CO_2_ reduction rates.^49^ This work showcases a promising noble metal‐free platform for the efficient photocatalytic conversion of CO_2_.

## 5 Conclusions and Outlook

In this review, we highlighted recent advancements in the field of Zn^+^‐related compounds and catalysts, including their synthesis, characterization and novel applications in energy conversion. The synthesis of monovalent Zn^+^ species is challenging, though such species can exist in Zn–Zn bonded organometallic compounds, confined zeolites systems or layered nanomaterials. Advanced characterization techniques, such as ESR, X‐ray structure analysis and UV–vis DR spectroscopy, allow monovalent Zn^+^ cations to be readily identified. DFT calculations are especially useful for exploring the formation mechanism of Zn^+^ species, and allow deep understanding of the role of Zn^+^ in electron transfer processes. Zn^+^‐containing heterogeneous catalysts and photocatalysts show great potential in energy conversion processes, enabling novel electron transfer pathways not realized in more common Zn^2+^ compounds.

In spite of the enormous recent progress in this field with synthetic methodologies and applications, there are many avenues for future research in Zn^+^ systems:

(1)   The reported synthetic approaches mainly focused on incorporating Zn^+^‐related cations in 3D supports (zeolites) and 2D supports (LDH systems). The limited number of each type of support restricts synthesis options. A possible future approach would be to synthesis Zn^+^ in other 2D layered materials, like Zn‐graphene or single/double Zinc chalcogenide. Such systems may allow the large‐scale and low‐cost synthesis of Zn^+^ species. In addition, combining Zn^+^ species and other coordinatively unsaturated metal ions such as Mg(I), Cu(I), Co(II) and Ti(III) in the same material, to produce “binary‐/ternary‐unsaturated metal center” catalysts may yield very active catalysts for many reactions.
(2)   From the viewpoint of characterization of Zn^+^ species, more information is required about the interconnectivity of such species with their support. Determination of the exact location and chemical environment of the Zn^+^ is a priority for better understanding the formation mechanism of such species, and also optimizing their performance as functional materials. In order to achieve this goal, more in situ techniques need to be developed that can probe Zn^+^. Techniques such as *in situ* EXAFS, *in situ* transient absorption spectra as well as *in situ* Fourier Transform Infrared (FTIR) spectrometry could all be applied to study Zn^+^ species formation and their roles in catalytic conversion processes. Such techniques could also be used to guide the rational design of more improved Zn^+^ catalysts.
(3)   With regard to applications, those highlighted above are mainly related to C1 chemistry. For example, the Zn^+^–V_o_ complex in LDH and Zn^+^‐supported ZSM‐5 catalysts show excellent activity toward CO_2_ photoreduction, conversion of methane and CO, and so forth. However, their activity and stability are still not high sufficient to demand consideration in practical applications. Through rational design, it may be possible to improve the catalytic activity of Zn^+^‐containing catalysts for CO_2_ and methane conversion, whilst eliminating deactivation under reaction conditions. It may also be possible to tune the catalyst structure to achieve CO_2_ reduction to high‐value >C_1_ hydrocarbons via an MTL pathway.

Over the next decade, Zn^+^ species are expected to attract increasing research attention, which could lead to important technological developments, particularly in the energy sector.
